# BK Virus Load Associated with Serum Levels of sCD30 in Renal Transplant Recipients

**DOI:** 10.1155/2016/9752097

**Published:** 2016-03-09

**Authors:** Haidar A. Shamran, Salma N. Malik, Jinan M. Al-Saffer, Rana S. Jawad

**Affiliations:** ^1^Medical research Unit, School of Medicine, University of Al-Nahrain, Baghdad 10066, Iraq; ^2^Medical Technical Institute, Baghdad 10066, Iraq; ^3^Biotechnology Department, School of Science, University of Baghdad, Baghdad 10066, Iraq; ^4^Biology Department, School of Science, University of Al-Mustansiriyah, Baghdad 10066, Iraq

## Abstract

*Background*. Rejection is the main drawback facing the renal transplant operations. Complicated and overlapping factors, mainly related to the immune system, are responsible for this rejection. Elevated serum levels of sCD30 were frequently recorded as an indicator for renal allograft rejection, while BV virus is considered as one of the most serious consequences for immunosuppressive treatment of renal transplant recipients (RTRs).* Aims*. This study aimed to determine the association of BK virus load with serum levels of sCD30 in RTRs suffering from nephropathy.* Patients and Methods*. A total of 50 RTRs with nephropathy and 30 age-matched apparently healthy individuals were recruited for this study. Serum samples were obtained from each participant. Real-time PCR was used to quantify BK virus load in RTRs serum, while ELISA technique was employed to estimate serum levels of sCD30.* Results*. Twenty-two percent of RTRs had detectable BKV with mean viral load of 1.094*E* + 06 ± 2.291*E* + 06. RTRs showed higher mean serum level of sCD30 (20.669 ± 18.713 U/mL) than that of controls (5.517 ± 5.304 U/mL) with significant difference. BK virus load had significant positive correlation with the serum levels of sCD30 in RTRs group.* Conclusion*. These results suggest that serum levels of sCD30 could be used as an indicator of BK viremia, and accordingly the immunosuppressive regime should be adjusted.

## 1. Introduction

Kidney transplantation is now considered the most appropriate choice for treatment of most patients suffering from end-stage renal disease (ESRD). However, rejection (acute or chronic) remains the most challenge facing the success of this maneuver. To overcome such challenge, immunosuppressive drugs have been successfully used but not without adverse effect. An effect gives a golden chance for opportunist microorganisms like polyomaviruses to exert their masked detrimental action inside the body [1]. Balanced using of immunosuppressive drugs may be a crucial factor in the follow-up of RTRs. However, even with high precaution taken in immunosuppressive using, a substantial portion of those patients develop progressive renal dysfunction and renal failure within a decade [2]. Therefore, prediction of renal rejection is of prior significance in these situations.

The CD30 molecule is 120-kDa transmembrane glycoprotein which is a member of tumor necrosis factor receptor (TNF-R) super family lacking a death domain [3]. The expression of this molecule in healthy tissues is limited to resting B and T lymphocytes [4, 5] and to less extent in activated monocytes and eosinophils [6]. Upregulation of CD30 occurs in activated lymphocytes in response to different stimuli. Many physiological functions have been proposed for CD30. Among these functions are the activation of both the c-Jun N-terminal kinase (JNK) and the nuclear factor *κ* B-cell (NF-*κ*B), and the production of reactive oxygen species (ROS) [7–9]. In addition, this molecule was found to have a costimulatory function in activation of lymphocyte especially Th2 cell, a function which promotes cytokine production as well as proliferation, differentiation, and survival of T cells [10, 11].

Following membrane expression of CD30 molecule, it is proteolytically cleaved to produce an 88-kDa soluble molecule in the body fluid [12]. Hence, serum levels of sCD30 can be used as a quantitative indicator for the cells expressing CD30. It has been found that serum levels of sCD30 correlated with the greater incidence of early onset of allograft rejection events [13], while elevated levels of sCD30 in the sera of patients on hemodialysis were reported from many studies [14, 15].

BK virus is a nonenveloped, double-stranded DNA virus of the polyomavirus family that primarily affects immunocompromised patients. BKV may cause nephropathy in renal transplant recipients receiving immunosuppressive therapy, resulting in renal dysfunction and, possibly, graft loss [16].

About 15% of RTRs have this virus in the absence of an effective strategy [17]. After primary infection, BK virus establishes a latency stage in the urinary epithelium and renal tubular epithelial cells. This study aimed to investigate the association of BK virus load with the serum levels of sCD30 in RTRs. Such correlation may be used clinically as a base to adjust the doses of immune suppressive drugs.

## 2. Materials and Methods

### 2.1. Study Population

A total of 50 RTRs with histopathologically confirmed nephropathy (based on the chronic interstitial fibrosis and tubular atrophy of the renal allograft) who were attending the Center of Kidney Diseases and Transplantation in the Medical City/Baghdad during the period from March 2014 to February 2015 were recruited for this study. The mean posttransplantation period was 16.134 months (less than 12 months in 29 patients and 12 months or more in 21 patients). From each participant, a consent form was obtained which includes information about the age, sex, and the date of transplantation. Exclusion criteria were delayed graft function, previous allograft, and history of episodes of infection during the first month after transplantation. Other 30 individuals (19 male and 11 female, mean age 47.68 years) were recruited as healthy control group. Anyone from this group who had a history of autoimmune disease or graft transplantation was excluded. Three mL of venous blood was collected from each patient in a plane tube where the serum was separated.

### 2.2. Immunological Assay

Enzyme-linked immunosorbent assay (Diagnostic Automation Inc., USA) was used to estimate serum levels of sCD30 using a commercially available kit (Invitrogen/USA) following manufacturer's instructions. Briefly, 150 *μ*L of distilled water (DW) was added to the standard and blank wells, while 140 *μ*L of DW was added to the sample wells. Sample (10 *μ*L) was added to designated wells, and the microplate was incubated at room temperature for 3 hrs and then washed three times. One hundred *μ*L of TMB substrate solution was added to each well and the microplate was incubated again at room temperature for about 15 min when the stop solution (100 *μ*L) was added to each well. The absorbance was read at 450 nm with spectrophotometer.

### 2.3. Estimation of BK Virus Load in Serum

Viral DNA was extracted from 400 *μ*L of serum using a ready kit (ExiPrep Viral DNA/RNA Kit, Bioneer, Korea) according to the manufacturer's instructions. Viral load was measured using AccuPower® BKV Quantitative PCR Kit, Bioneer, Korea, which detects and quantitates the four genotypes of BKV DNA in human serum with quantitative range of 150–10^7^ copies/mL. A region within the BKV small t-antigen gene, which encodes the capsid proteins (VP1, VP2, and VP3) and the agnoprotein, was amplified using Exicycler*™* 96 thermocycler (Bioneer, Korea). Internal positive control (IPC) was used to check whether PCR is inhibited by the sample and to determine the amplification of nucleic acids in each well.

Nontemplate control was used to determine whether the sample is contaminated in the process of sample pretreatment, nucleic acid extraction, and PCR preparation. Taqman probe with FAM and TAMRA as fluorophore and quencher, respectively, was employed for the detection of PCR product. The real-time data was collected at the second step of the amplification cycle.

**Figure 1:**
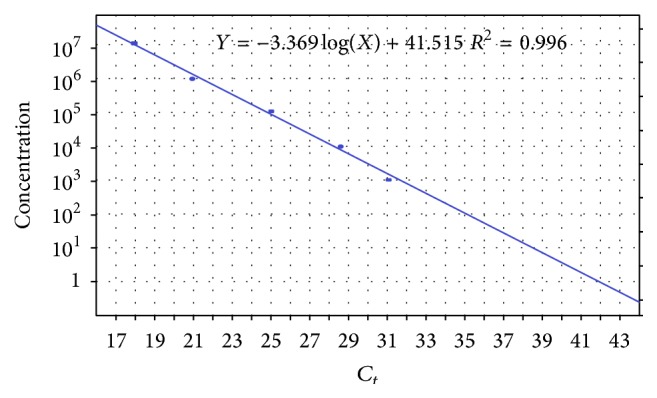
Standard curve. The five standards are 10^3^, 10^4^, 10^5^, 10^6^, and 10^7^ copies/mL.

A standard curve (Figure 1) was created using five quantitated BKV DNA controls ranging from 1 × 10^3^ copies/mL to 1 × 10^7^ copies/mL. The thermal protocol included initial denaturation at 95°C for 10 min followed by 50 cycles of denaturation at 95°C for 30 sec and annealing and synthesis at 54°C for 90 sec, with final holding stage at 25°C for up to 1 hr.

### 2.4. Statistical Analysis

Statistical package for social sciences version 16.0 (Chicago, USA) was used to analyze the data. Values were expressed as mean ± standard deviation (SD). Independent sample *t*-test was used to compare mean sCD30 serum levels between RTRs and control, while bivariate correlation test was employed to examine the correlation between BKV loads and serum levels of sCD30. Odds ratio for different risk factors was calculated using logistic regression test. The acceptable level of significant was *P* value ≤ 0.05.

## 3. Results

This study included 50 RTRs with confirmed nephropathy and 30 individuals as healthy control group.  Table 1 shows demographic and clinical features of the nephropathic patients.

Out of 33 RTRs whose ages are less than 40 years, only 4 (12.12%) were positive for BKV compared to 7 (41.11%) out of 17 RTRs whose ages are ≥40 years with significant difference (OR = 5.075, 95% CI = 1.223–21.065) (Table 2). Exactly one-quarter (25%) of male RTRs were positive for BKV compared to 14.28% of female. However, the difference was nonsignificant (OR = 0.5, 95% CI = 0.094–2.673). RTRs whose PTP was less than 12 months showed higher percentage of BK viremia (27.58%) than those those whose PTP was 12 months or longer, but the difference was nonsignificant (OR = 0.348, 95% CI = 0.101–1.90) (Table 2).

### 3.1. BKV Load

Out of 50 enrolled RTRs, 11 (22%) showed detectable BK viremia with viral load which ranged from 6.12*E* + 04 to 7.81*E* + 06, mean = 1.098*E* + 06 ± 2.291*E* + 06, while none of the control group was positive for BK viremia (Table 3).

### 3.2. Serum Levels of sCD30

**Figure 2:**
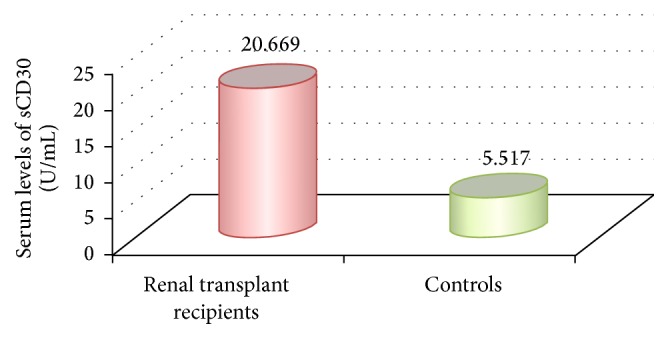
Mean serum level of sCD30 in renal transplant recipients and controls.

Renal transplant recipients showed relatively high levels of sCD30 which ranged from 2.252 U/mL to 97.144 U/mL, mean = 20.669 ± 18.713 U/mL, compared to that of controls which ranged from 0.291 U/mL to 18.242 U/mL, mean = 5.517 ± 5.304 U/mL, with highly significant difference (*t* = 4.322, *P* = 0.004) (Figure 2).

### 3.3. Correlation between BKV Loads and Serum Levels of sCD30 in RTRs

**Figure 3:**
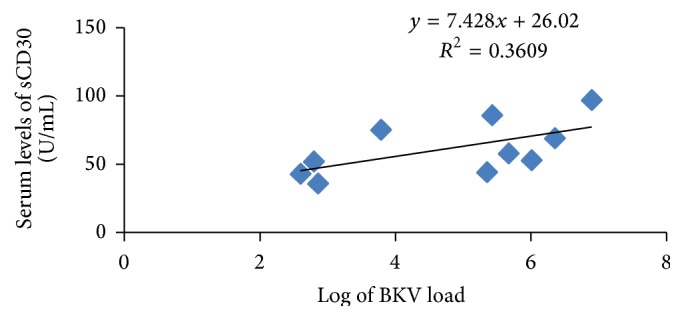
The association between sCD30 and BKV load.

All serum sample positive for BKV had serum level of sCD30 beyond the normal limit (20 U/mL). Correlation test revealed positive significant correlation between the log of BKV loads and serum levels of sCD30 (*r* = 0.694, *P* = 0.018) (Figure 3).

## 4. Discussion

The quantification of BK virus load in blood is a useful tool not only for diagnosis of BKV nephropathy but also for monitoring the response to the therapy. When this quantification is associated with estimation of serum levels of sDC30, it can give valuable information to decide how to deal with the immunosuppressive regime that is given for the patients and represent a prognostic factor for the possible graft rejection.

The only significant risk factor for BKV in this study was patient's age. In fact, it is unfair to exclude the other factors which appeared nonsignificant perhaps due to the relative small size of the sample. Anyhow, it is well documented that older age is a risk factor for BKV [17]. It is logical to explain this by the impaired immune response in the older ages. The influences of aging on immune system are very extensive and involve a reduction in the rate at which naïve T and B cells are introduced. Besides, aging affects the composition and quality of the mature lymphocyte [18].

The result of the current study revealed that 22% of the RTRs are positive for BKV. This result disagreed with that of Al-Obaidi et al. [19] who detected BK viremia in only 12.1% in Iraqi RTRs. This difference can be attributed to the analytic sensitivity of kit used for quantification of the viral DNA which is 800 copies/mL compared with 150 copies/mL for our kit. Furthermore, the target gene can also influence the result. The kit used in our study targets small t-antigen gene, while the kit used by Al-Obaidi et al. targets large t-antigen [19]. However, as high as 31% of BK viremia was recorded in a cohort study of Greek RTRs patients [16]. Generally, variations in sample type, DNA extraction techniques, primers and probe sequences, and BKV strain DNA used for standard curve creation can all influence the quantification results and introduce clinically significant variability [20]. It is worth mentioning that the constitutive evaluation of viremia was not conducted neither in the aforementioned studies nor in ours. Accordingly BKV load may not reflect the actual viremia in the whole kidney transplant population.

There is no consensus about the cut-off viral load of BKV which could be considered of clinical importance, although a retrospective study has suggested that a BK virus load > 4 log copies/mL is strongly associated with finding BKV on biopsy [21]. However, detection of BKV in whatever quantity in blood components (plasma or serum) by real-time PCR is very sensitive and specific. Sensitivity and specificity in such case are 100% and about 90%, respectively [22]. That is because, in order for the virus to be reactivated and reproduced in blood, it has been already reproduced and probably caused some lesions in tubular epithelial cells of the kidney where it was in latent state [23]. Therefore, using quantitative PCR kit which can detect relatively low viral load is of crucial importance for screening tests.

In many cases, viral infections could result in an elevation in sCD30. For instance, Fattovich et al. [24] demonstrated that serum levels of sCD30 have increased in most patients with HBs Ag-positive chronic hepatitis, while Haque et al. [25] found that such increase was associated with infection with Epstein-Barr Virus (EBV). Thus, it is not unreasonable to find high levels of sCD30 associated with BKV. But this situation implies a dilemma because our patients are supposed to be under immune suppression and the overall immune cells (including that bearing CD30) are reduced.

To explain this discrepancy, we must keep in mind that immune suppressors skew the balance in favor of graft tolerance by promoting the regulatory (Foxp3+, CD4+, and CD25+) T cells while inhibiting the cytotoxicity of T effector cells [26]. Thus, the number of CD30-bearing cells will not be affected by this treatment. On the other hand, viral infection can cause activation of immune cells, especially CD8+ T lymphocytes. This activation is not necessarily associated with the increased number of these cells. Rather, there will be a stimulation of proteolytic cleavage of CD30 followed by the release of sCD30 into the blood stream. Although the mechanism of this cleavage is not clear, the metalloproteinase enzyme may have a role. The positive correlation between BK virus load and serum levels of sCD30 supports this hypothesis. Thus, it seems that sCD30 is not necessarily an indicator of the intensity of the immune response because cleavage of CD30 could be induced even with the presence of fixed number of immune cells.

Elevated serum levels of sCD30 in RTRs were recorded in many previous studies and were used as a bad prognostic factor for graft rejection [27, 28], because it indicates cellular and/or humeral arms of immune system against the allograft. Such activation is accompanied by increase in the absolute numbers of CD30-bearing cells. Then, the cleavage of this marker by whatever cause leads to increase in the serum level of sCD30.

One limitation of this study is that both BKV load and sCD30 levels were only evaluated once due to technical and funding difficulties. It is expected that BKV load undergoes fluctuations with the time influenced by host's immunity status. On the other hand, sCD30 levels do not just reflect the activation of immunity against the virus but may also reflect the activation of alloreactive cells. Thus, performing serial measurements of sCD30 and BKV load can give better evaluation of sCD30 as a sensible biomarker for BKV infection in RTRs with nephropathy.

However, the results of the present study positively suggest that BKV associates with an elevation in the serum levels of sCD30, and this marker can be used as an indicator not only for the risk of graft rejection but also for the possible replication of BKV.

## Figures and Tables

**Table 1:** Demographic and clinical features of the nephropathic patients.

Variable	Value
Age (mean ± SD)	48.14 ± 12.7
Sex M : F (No)	36 : 14
*Clinical features (No, %)*	
Proteinuria	48 (96%)
Hypertension	21 (42%)
Hematuria	32 (62%)
Anemia	12 (24%)
Thrombotic microangiopathy	6 (12%)
Dyslipidemia	28 (56%)
Glomerulonephritis	3 (6%)
Chronic pyelonephritis	1 (2%)
Diabetic nephropathy	3 (6%)
Renal amyloidosis	2 (4%)
Preeclampsia	1 (2%)
Obstructive uropathy	1 (2%)
*Donors (No, %)*	
Related	38 (76%)
Unrelated	12 (24%)

**Table 2:** Risk factor for infection with BKV.

Risk factor	Total number for positive cases = 11	OR (95% CI)
Age		
<40 years (33)	4 (12.12%)	1.0
≥40 years (17)	7 (41.11%)	5.075 (1.223–21.065)
Gender		
Male (36)	9 (25%)	1.0
Female (14)	2 (14.28%)	0.5 (0.094–2.673)
Posttransplantation period		
<12 months (29)	8 (27.58%)	1.0
≥12 months (21)	3 (14.28%)	0.348 (0.101–1.90)

OR = odds ratio and CI = confidence interval.

**Table 3:** BK virus load in renal transplant recipients.

Well	Sample ID	IPC result	BKV *C*_*t*_	BKV (copy/mL)	BKV result
A1	NTC	Valid	Undetermined	—	Valid
B1	SPC1	Valid	31.04	1.0*E* + 03	Valid
C1	SPC2	Valid	28.42	1.0*E* + 04	Valid
D1	SPC3	Valid	25.00	1.0*E* + 05	Valid
E1	SPC4	Valid	20.97	1.0*E* + 06	Valid
F1	SPC5	Valid	17.92	1.0*E* + 07	Valid
G1	Sample 01	Valid	—	—	Not Detected
H1	Sample 02	Valid	31.89	7.2*E* + 02	7.2*E* + 02
A2	Sample 03	Valid	—	—	Not detected
B2	Sample 04	Valid	—	—	Not detected
C2	Sample 05	Valid	—	—	Not detected
D2	Sample 06	Valid	—	—	Not detected
E2	Sample 07	Valid	28.76	6.12*E* + 03	6.12*E* + 03
F2	Sample 08	Valid	—	—	Not detected
G2	Sample 09	Valid	—	—	Not detected
H2	Sample 10	Valid	—	—	Not detected
A3	Sample 11	Valid	—	—	Not detected
B3	Sample 12	Valid	23.24	2.66*E* + 05	2.66*E* + 05
C3	Sample 13	Valid	—	—	Not detected
D3	Sample 14	Valid	—	—	Not detected
E3	Sample 15	Valid	—	—	Not detected
F3	Sample 16	Valid	—	—	Not detected
G3	Sample 17	Valid	—	—	Not detected
H3	Sample 18	Valid	—	—	Not detected
A4	Sample 19	Valid	32.76	3.98*E* + 02	3.98*E* + 02
B4	Sample 20	Valid	—	—	Not detected
C4	Sample 21	Valid	—	—	Not detected
D4	Sample 22	Valid	—	—	Not detected
E4	Sample 23	Valid	—	—	Not detected
F4	Sample 24	Valid	18.29	7.81*E* + 06	7.81*E* + 06
G4	Sample 25	Valid	—	—	Not detected
H4	Sample 26	Valid	—	—	Not detected
A5	Sample 27	Valid	—	—	Not detected
B5	Sample 28	Valid	—	—	Not detected
C5	Sample 29	Valid	32.1	6.24*E* + 02	6.24*E* + 02
D5	Sample 30	Valid	—	—	Not Detected
E5	Sample 31	Valid	22.42	4.67*E* + 05	4.67*E* + 05
F5	Sample 32	Valid	—	—	Not detected
G5	Sample 33	Valid	—	—	Not detected
H5	Sample 34	Valid	—	—	Not detected
A6	Sample 35	Valid	—	—	Not detected
B6	Sample 36	Valid	—	—	Not detected
C6	Sample 37	Valid	—	—	Not detected
D6	Sample 38	Valid	20.12	2.24*E* + 06	2.24*E* + 06
E6	Sample 39	Valid	—	—	Not detected
F6	Sample 40	Valid	21.27	1.02*E* + 06	1.02*E* + 06
G6	Sample 41	Valid	—	—	Not detected
H6	Sample 42	Valid	—	—	Not detected
A7	Sample 43	Valid	—	—	Not detected
B7	Sample 44	Valid	23.48	2.25*E* + 05	2.25*E* + 05
C7	Sample 45	Valid	—	—	Not detected
D7	Sample 46	Valid	—	—	Not detected
E7	Sample 47	Valid	32.2	5.82*E* + 02	5.82*E* + 02
F7	Sample 48	Valid	—	—	Not detected
G7	Sample 49	Valid	—	—	Not detected
H7	Sample 50	Valid	—	—	Not detected
